# Functional genomics analysis of a phyllospheric *Pseudomonas spp* with potential for biological control against coffee rust

**DOI:** 10.1186/s12866-022-02637-4

**Published:** 2022-09-22

**Authors:** Leandro Pio de Sousa, Matheus Aparecido Pereira Cipriano, Marcio José da Silva, Flávia Rodrigues Alves Patrício, Sueli dos Santos Freitas, Marcelo Falsarella Carazzolle, Jorge Maurício Costa Mondego

**Affiliations:** 1 Instituto Agronômico de Campinas, IAC, Campinas, SP Brazil; 2 Programa de Pós-Graduação Em Genética E Biologia Molecular, UNICAMP, Campinas, SP Brazil; 3 UNICAMP, CBMEG, Campinas, SP Brazil; 4 Instituto Biológico, Campinas, SP Brazil; 5 Instituto de Biologia, Laboratório de Genômica E Expressão, UNICAMP, Campinas, SP Brazil

**Keywords:** Pseudomonas, Phyllosphere, Genomics, Coffee rust, Biocontrol, Coffee

## Abstract

**Background:**

*Pseudomonas spp.* promotes plant growth and colonizes a wide range of environments. During the annotation of a *Coffea arabica* ESTs database, we detected a considerable number of contaminant *Pseudomonas* sequences, specially associated with leaves. The genome of a *Pseudomonas* isolated from coffee leaves was sequenced to investigate in silico information that could offer insights about bacterial adaptation to coffee phyllosphere. In parallel, several experiments were performed to confirm certain physiological characteristics that could be associated with phyllospheric behavior. Finally, in vivo and in vitro experiments were carried out to verify whether this isolate could serve as a biocontrol agent against coffee rust and how the isolate could act against the infection.

**Results:**

The isolate showed several genes that are associated with resistance to environmental stresses, such as genes encoding heat/cold shock proteins, antioxidant enzymes, carbon starvation proteins, proteins that control osmotic balance and biofilm formation. There was an increase of exopolysaccharides synthesis in response to osmotic stress, which may protect cells from dessication on phyllosphere. Metabolic pathways for degradation and incorporation into citrate cycle of phenolic compounds present in coffee were found, and experimentally confirmed. In addition, MN1F was found to be highly tolerant to caffeine. The experiments of biocontrol against coffee leaf rust showed that the isolate can control the progress of the disease, most likely through competition for resources.

**Conclusion:**

Genomic analysis and experimental data suggest that there are adaptations of this *Pseudomonas* to live in association with coffee leaves and to act as a biocontrol agent.

**Supplementary Information:**

The online version contains supplementary material available at 10.1186/s12866-022-02637-4.

## Background

Coffee (*Coffea arabica* L) is one of the most consumed brewed drink in the world, being one of the most valued commodities on the market [1]. The coffee production chain has a great socio-economic impact, moving around US$ 90 billion a year and involving around 500 million people worldwide, including producers, rural workers, traders and coffee producers [2]. Although more than 100 species of the genus *Coffea* have been described, only *Coffea arabica* and *Coffea canephora* are effectively cultivated and commercialized. *C. arabica* is an autogamous allotetraploid plant, the result of a recent cross between *C. canephora*, or related species, and *C. eugenioides* [3]. Such autogamy leads to a narrow genetic basis, aggravated by the fact that most of the *C. arabica* planted today have only two varieties as base populations, Typica and Bourbon [4]. Because of this narrow genetic basis, *C. arabica* plantations are quite susceptible to diseases, especially coffee rust.,caused by the biotrophic fungus *Hemileia vastatrix* [5]. Traditionally, disease control is based on copper-based pesticides, but this practice has been restricted by the new environmental guidelines [6]. Thus, biological control has been a good alternative for the control of coffee rust, especially in organic cultivation [7].

Bacteria of the genus *Pseudomonas* comprise a diverse group of microorganisms that can be isolated from different environments including fresh water, salt water, soil, internal tissues of plants, and food [8]. Investigation about *Pseudomonas* spp. associated with plants leads to great interest because of their benefits on plant host by producing auxins and siderophores, which improve plant growth and development by increasing yield, mediating resistance against other pathogens and providing competitive advantages [9]. As a result, these bacteria have been recognized as potential tools for the development of biofertilizers and biocontrol products for agriculture [10].

*Pseudomonas* have been detected in coffee tissues [11]. Moreover, during annotation of the coffee expressed sequence tag (EST) database for the Brazilian Coffee Genome Project (BCGP; [4]), *Coffea arabica* was found to contain a large amount of ‘contaminant’ bacterial sequences on the surface of leaves, most of which belonged to *Pseudomonas* spp.. In this study, we report a new biocontrol strain of *Pseudomonas (Pseudomonas sp.* MN1F*)*, which was isolated from coffee leaves, and analyzed experimental and genomic data through two perspectives: 1) the adaptive mechanisms that allow survival on the leaf surface; 2) the possible effect as a biocontrol agent against coffee rust.

## Results

### Identification and genomic information

The isolate named *Pseudomonas* sp. MN1F was confirmed as belonging to *Pseudomonas* genus with proximity to *Pseudomonas putida* and *Pseudomonas monteilii* (Figure S1). Bacterial sequencing was performed using Illumina technology. After trimming and assembly, genome length was estimated at 6 Mbp, consistent with other *Pseudomonas* sequenced genomes [12, 13]. C + G content, number of coding sequences and number of tRNAs were 62,9%, 5.575 and 45, respectively. Global genomes statisitics are depicted in Table 1. Forty-eight insertion sequences (IS) were found, 34 of which were similar to *Pseudomonas syringae* sequences (Table S1).

**Table 1:** Global statistics of Pseudomonas MN1F genome

Total sequence length	6,075,282
Total ungapped length	6,075,282
Number of contigs	79
Contig N50	125,670
Contig L50	15
Total number of chromosomes and plasmids	0
Genes (total)	5,730
CDS (total)	5,681
Genes (coding)	5,575
CDS	5,575
tRNAs	45
Non coding RNAs	4

Gene clusters of secondary metabolites were analyzed using the antiSMASH [14], indicating that MN1F has 31 genes related to pyoverdin synthesis, expression and transport. Genes related to antibiotic resistance beta-lactamase (1 gene) and efflux pumps (7 genes) were found. Numerous genes related to abiotic stresses have also been found, such as temperature stress (5 heat-shock proteins, 5 cold-shock proteins and 7 universal stress proteins), osmotic stress (1 Aquaporin Z, 7 proline/betaine transporter, 1 glycerol uptake protein, 1 osmotically inducible protein), oxidative stress (4 peroxidase, 1 metallothionein and 6 superoxide dismutase), amino acid and carbon starvation (*VapBC* toxin/anti-toxin system, 3 carbon starvation proteins). Our annotation revealed genes related to alginate synthesis (*Alg8, Alg44, AlgK, AlgE, AlgJ, AlgF, AlgA, AlgL, AlgT* and *AlgX*), which may contribute for exopolysachharides (EPS) formation. Genes for sigma fimbriae (usher protein, adhesion and chaperone), for type I (*LapB, C, D, E, P*, and *RTX*) and type VI (*IcmF, B, G, A, VasA, F, B, D, I, VCA0109* and *VgrG*) secretion systems were also detected. In addition, five copies for rearrangement hotspot (rhs) repeat proteins were found.

**Fig. 1:**
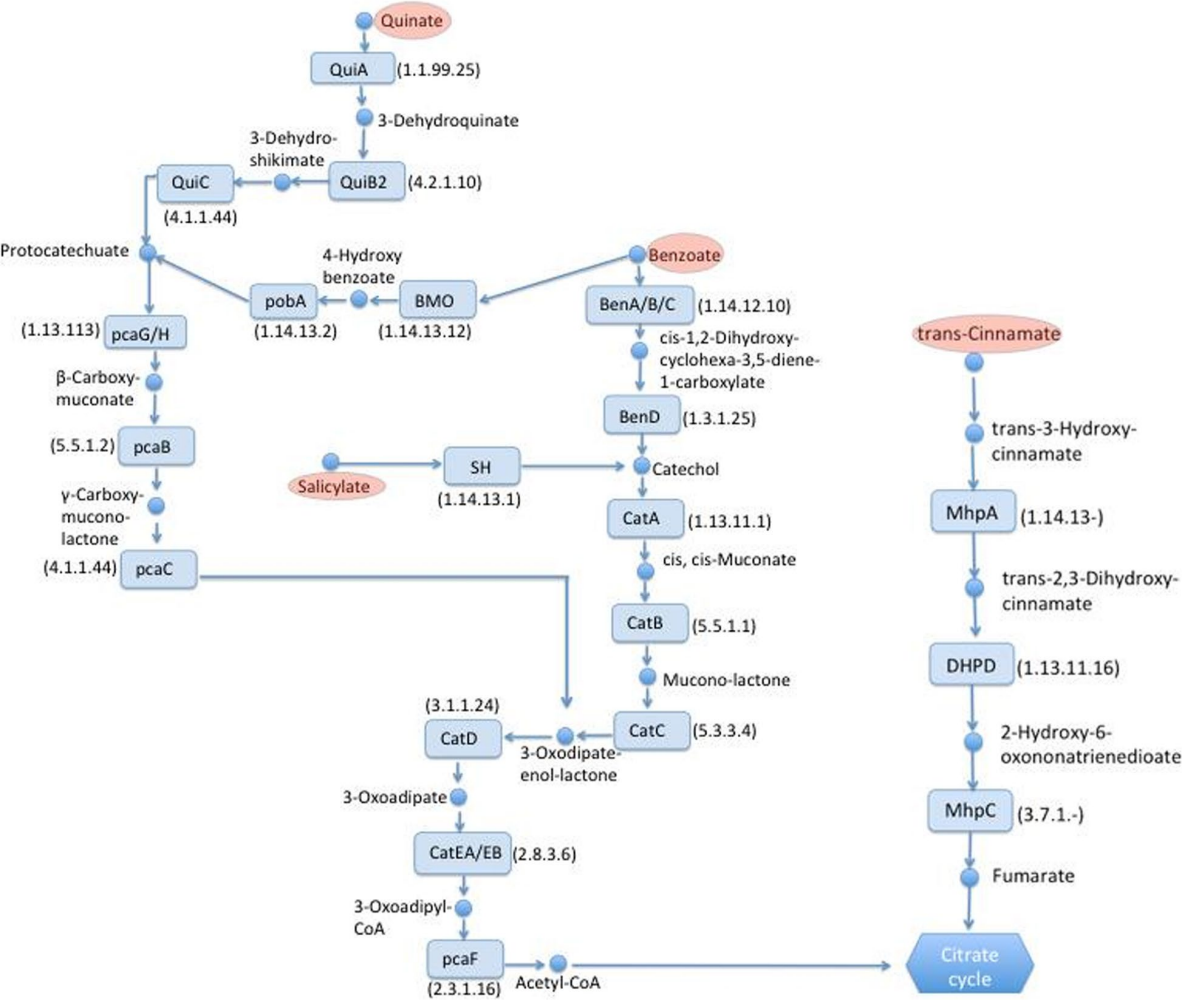
Metabolic map of pathway interconnections between quinate, benzoate, trans-cinnamate and salicylate catabolic systems. Precursors are indicated in red, enzymes are indicated in blue squares and intermediate compounds are indicated in small blue circles. Drawing based on Kyoto Encyclopedia of Genes and Genomes (KEGG) homepage (https://www.genome.jp/kegg)

**Fig. 2:**
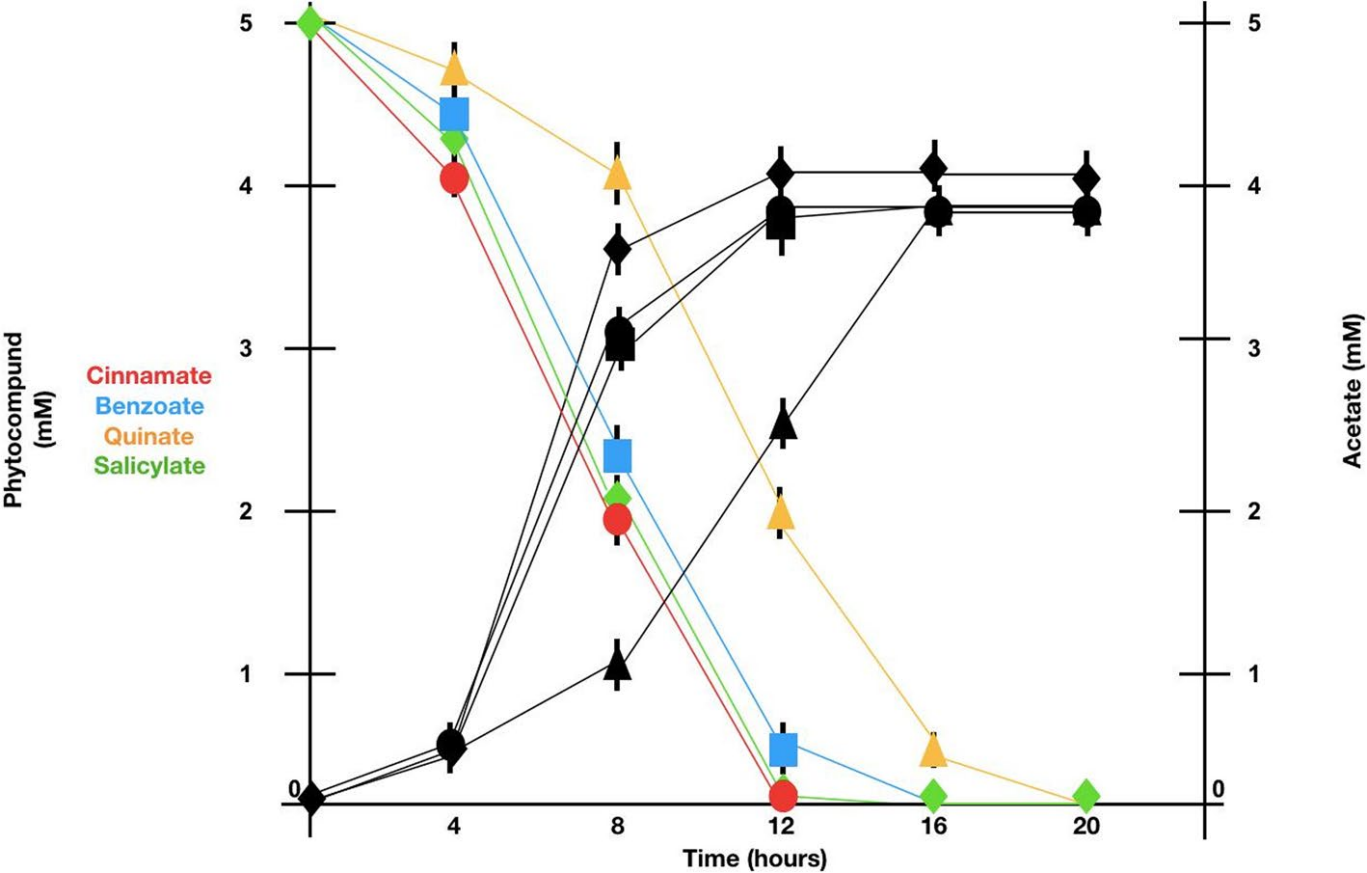
Measurement of phytocompounds incorporation into citrate cycle. Incorporation was measured by the decreasing concentration of phytocompounds (Coloured lines: cinnamate: red circle, benzoate: blue square, quinate: yellow triangle, salicylate: green rhombus) and concomitant acetate synthesis (Black lines: cinnamate: black circle, benzoate: black square, quinate: black triangle, salicylate: black rhombus)

In order to evaluate whether MN1F uses coffee molecules as energy sources, genes that are involved in the degradation of phytocompounds were searched. MN1F possess the genetic machinery required for the degradation of benzoate, quinate, cinnamate, and salicylate; phenolics commonly found in coffee plants [15–17]. The interconnection between these degradation systems culminates with the formation of acetyl-CoA, a precursor for citrate synthesis (Fig. 1). The degradation pathways of benzoate and salicylate converge on the conversion into catechol by *BenD* and *SH* enzymes, respectively. The quinate pathway converges on the benzoate pathway through the formation of 3-oxoadipate-enol-lactone, but the reverse may also occur when the benzoate pathway (via 4-hydroxy benzoate) converges to the quinate pathway. These three pathways end at acetyl-CoA. The cinnamate pathway, however, does not interconnect with any other and is incorporated into the citrate cycle, ending at fumarate rather than acetyl-CoA. HPLC experiments showed that MN1F can actually break the previous cited phytocompounds and incorporate them into the citrate cycle. Such incorporation was measured by the increasing of concentration of acetate by using the phytocompounds benzoate, cinnamate, quinate and salycilate as unique carbon source (Fig. 2).

### EPS production and caffeine resistance

**Fig. 3:**
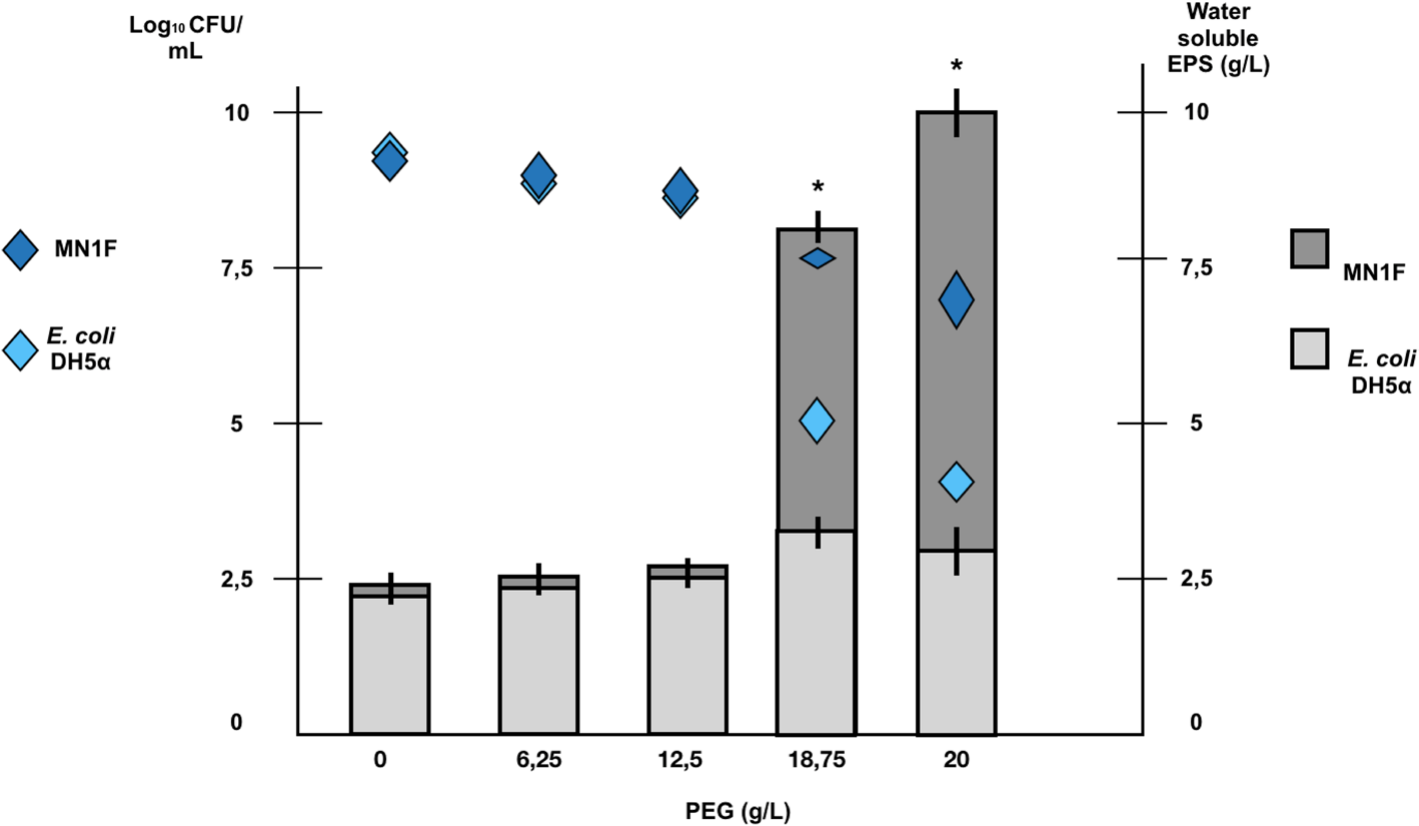
Production of water-soluble exopolysaccharides (EPS) by MN1F in response to PEG concentration. Bars represent water soluble EPS (g/L); dark gray bars: MN1f; light gray bars: *E. Coli*. Rhombus represent CFU (Log_10_ CFU/mL); dark blue rhombus: MN1F; light blue rhombus: *E. coli*. The asterisk indicates statistically significant differences (Tukey, α = 0.05)

Exopolysaccharides (EPS) are involved in regulating mechanisms that promote tolerance to desiccation in bacteria and fungi [18]. Based on the fact that leaf surface is an environment that is poor in water [19, 20], EPS production at increasing osmotic pressure was measured during MN1F growth at minimal media plus PEG. MN1F responded to induced drought stress by producing large amounts of EPS (Fig. 3; dark grey, > 10 g/L at high PEG concentrations).

After extensive genome annotation, no genes were found for caffeine degradation. To evaluate whether MN1F could incorporate caffeine by a non-canonical pathway, we grew bacteria in media with glucose (0—2 g) and NH_4_Cl (0—8 g) as carbon and nitrogen primary sources, respectively. The intention was to check whether MN1F induce cells to “replace” glucose and NH_4_Cl with caffeine. MN1F was not able to grow in media containing caffeine in low concentrations of caffeine (Fig. S2).

**Fig. 4:**
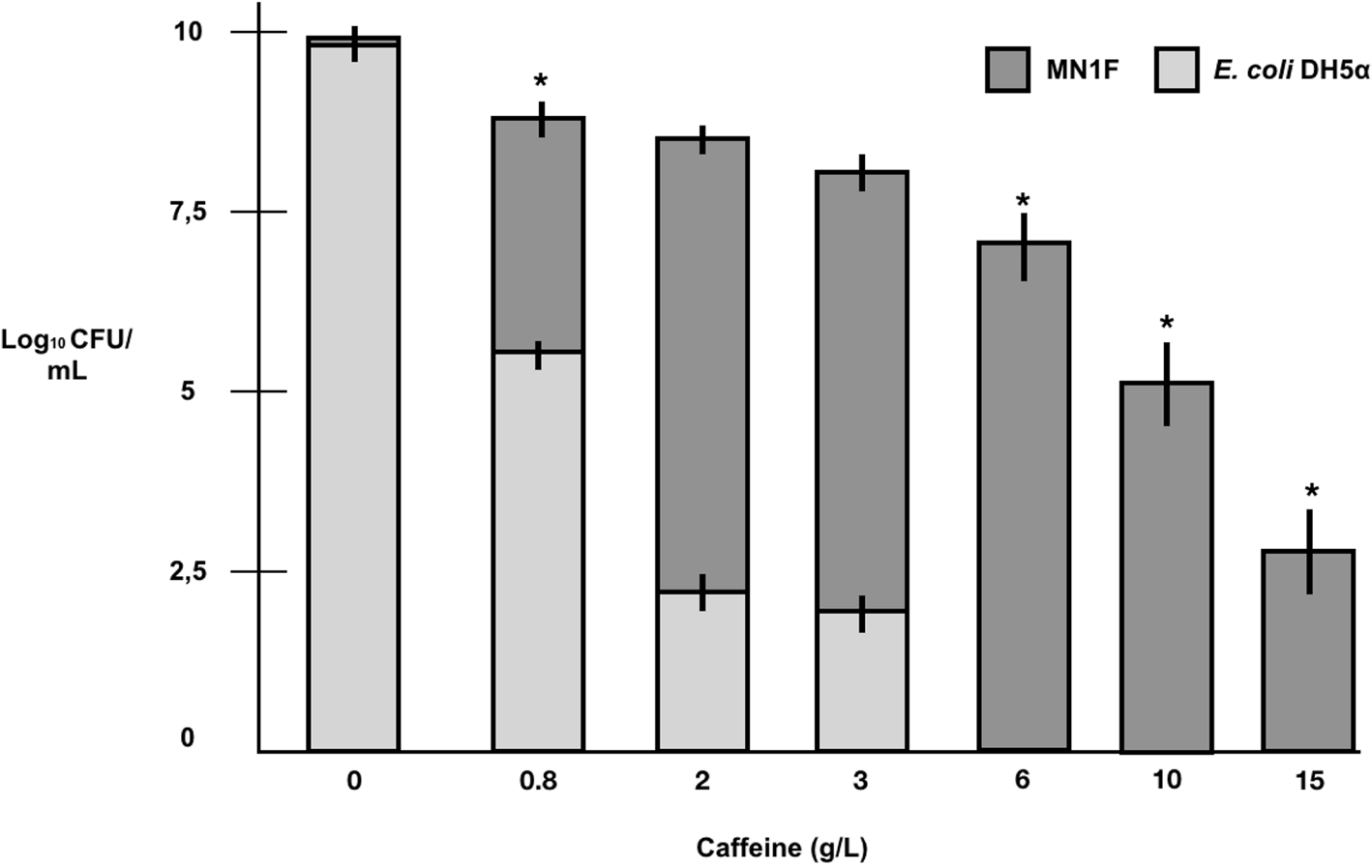
Evaluation of caffeine resistance in MN1F. Dark gray bars: MN1F; Light gray bars: *E. coli* negative control. Bacteria growth was measured by CFU (Log_10_ CFU/mL). The asterisk indicates statistically significant differences (Tukey, α = 0.05)

We evaluated MN1F resistance to high concentrations of caffeine (Fig. 4). Different caffeine concentrations were used (0.8, 2, 3, 6, 10, 15 g/mL) and measured bacterial growth after 8 h of incubation. MN1F was more resistant to caffeine than *E. coli* control (Fig. 4), indicating some degree of resistance to caffeine.

### Biocontrol against coffee leaf rust

**Fig. 5:**
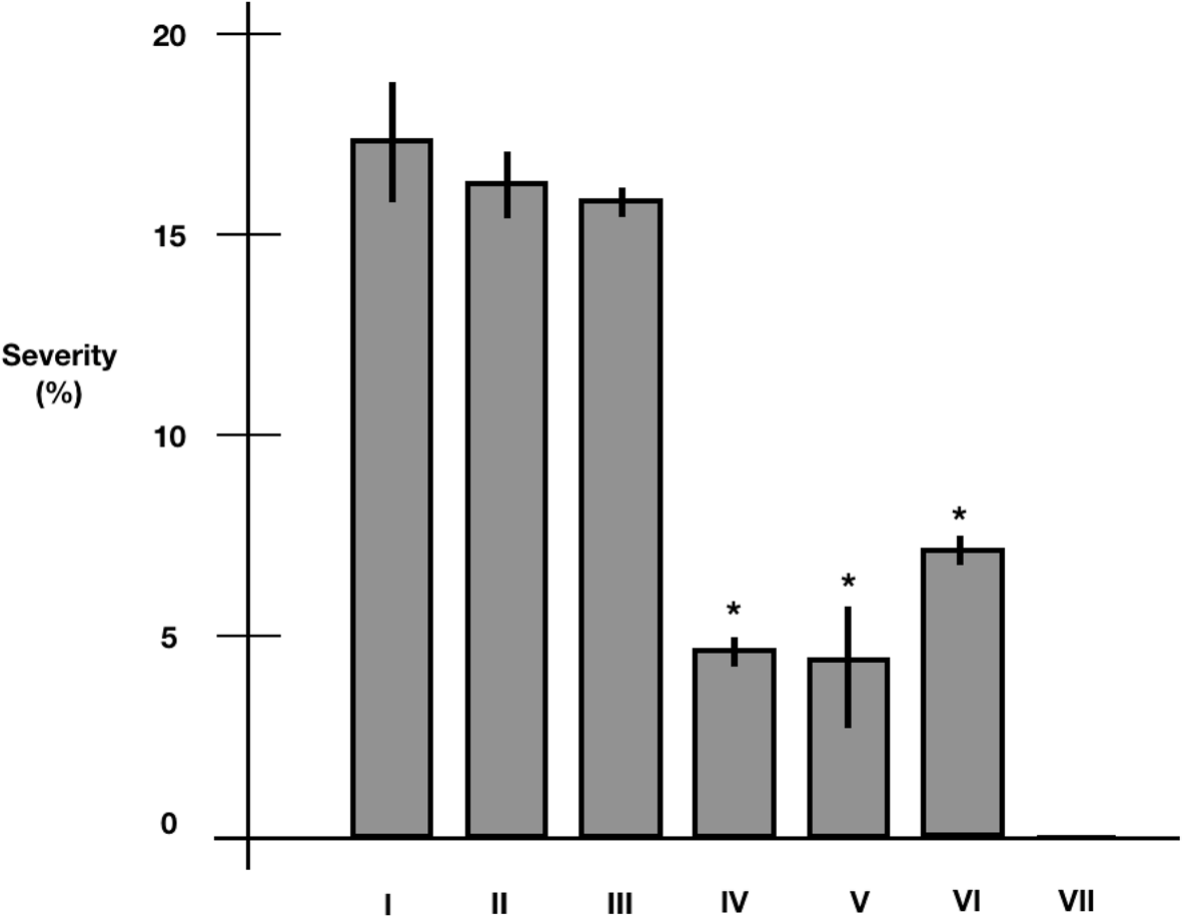
Severity of *H. vastatrix* infection after MN1F application. I- saline + uredospores applied simultaneously; II—King’s B liquid medium + uredospores applied simultaneously; III – MN1F supernatant filtered + uredospores applied simultaneously; IV—MN1F + uredospores applied simultaneously; V—MN1F + uredospores applied 24 h later; VI—Uredospores + MN1F applied 24 h later; VII—MN1F only; The asterisk indicates statistically significant differences (Dunnett, *p* < 0.05)

The presence of Type I and VI secretion systems in MN1F gave us a hint that this bacteria could be used as a biocontrol agent. An initial experiment was performed to check whether MN1F had an antagonistic effect on *H. vastatrix* infection in *C. arabica* detached leaves (Figure S3). The application of MN1F decreased the severity of the infection (Fig. 5; treatment IV, V, VI). However, MN1F was less effective when applied after 24 h of *H. vastatrix* uredospores application (Fig. 5; treatment VI). It was verified whether the antagonism was due to extracellular metabolites secreted by MN1F. The supernatant was collected, filtered, and applied to the leaves with the uredospores. The severity of the infection did not differ between the application of the supernatant (Fig. 5; treatment III), the control treatments with saline (Fig. 5; treatment I) and culture medium (Fig. 5; treatment II).

**Fig. 6:**
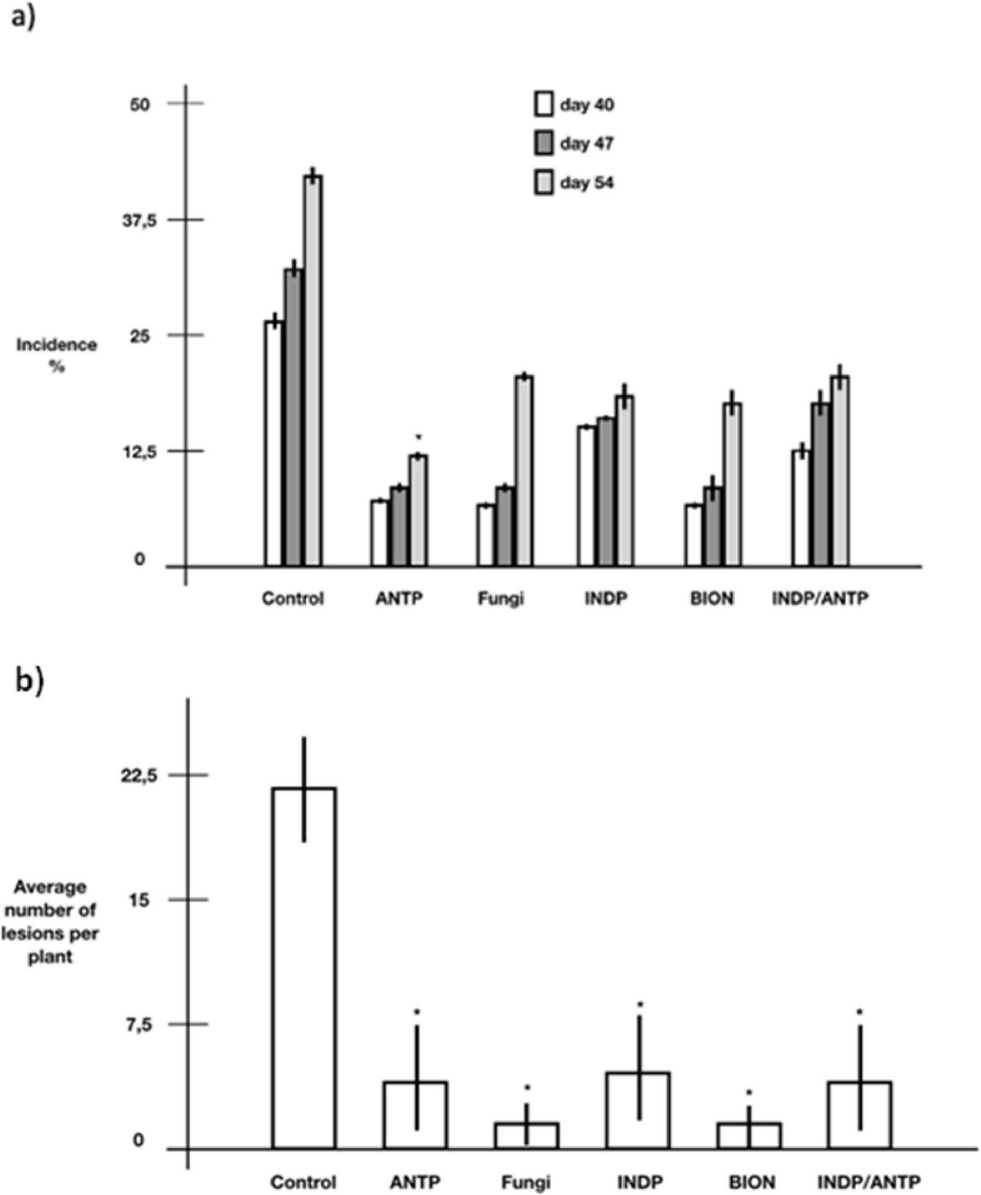
In vivo antagonism of MN1F against *H. vastatrix.* a) Incidence of coffee rust in response to MN1F and other treatments. Control—Infected plants without treatment. BION (ASM)—plants sprayed with ASM (0.01 g/L) two days before infection; Fungi (fungicide Priori Xtra)—plants sprayed with Priori fungicide (50% v/v) two days before infection; INDP—plants sprinkled with MN1F two days before infection; ANTP—plants sprinkled with MN1F few minutes before infection; ANTP/INDP—plants that were sprinkled with MN1F two days before and a few minutes before infection; b) Severity, evaluated by the average number of lesions per plant in the same experiment; at 54 days after inoculation (Dunnett, *p* < 0.05)

In vivo antagonism was verified by calculating the *H. vastatrix* incidence in response to MN1F application (Fig. 6a). The treatments with the fungicide azoxistrobin + cyproconazole (Fungi), the resistance inducer acibenzolar-S-methyl (ASM) and MN1F inoculant showed a positive effect in delaying the progress of infection, since all had a lower incidence than control plants. MN1F treatments ANTP (antagonism), INDP (induction) and INDP/ANTP (antagonism and induction) showed a final effect similar to the fungicide and ASM. However, after the final measurement, the application of MN1F few minutes before infection (ANTP) showed the best result of all treatment with an incidence of 12%, a better result than chemical treatments with 18% of incidence. Treatment with application two days and few minutes before infection (INDP/ANTP) proved to be inferior (around 18% of incidence) to ANTP alone. In addition. treatment with MN1F also reduced the average number of lesions per plant by about fourfold (Fig. 6b) compared to the untreated control, but there was no significant difference (*p* > 0.05) between treatment with MN1F and the other treatments (with BION and fungicide).

**Fig. 7:**
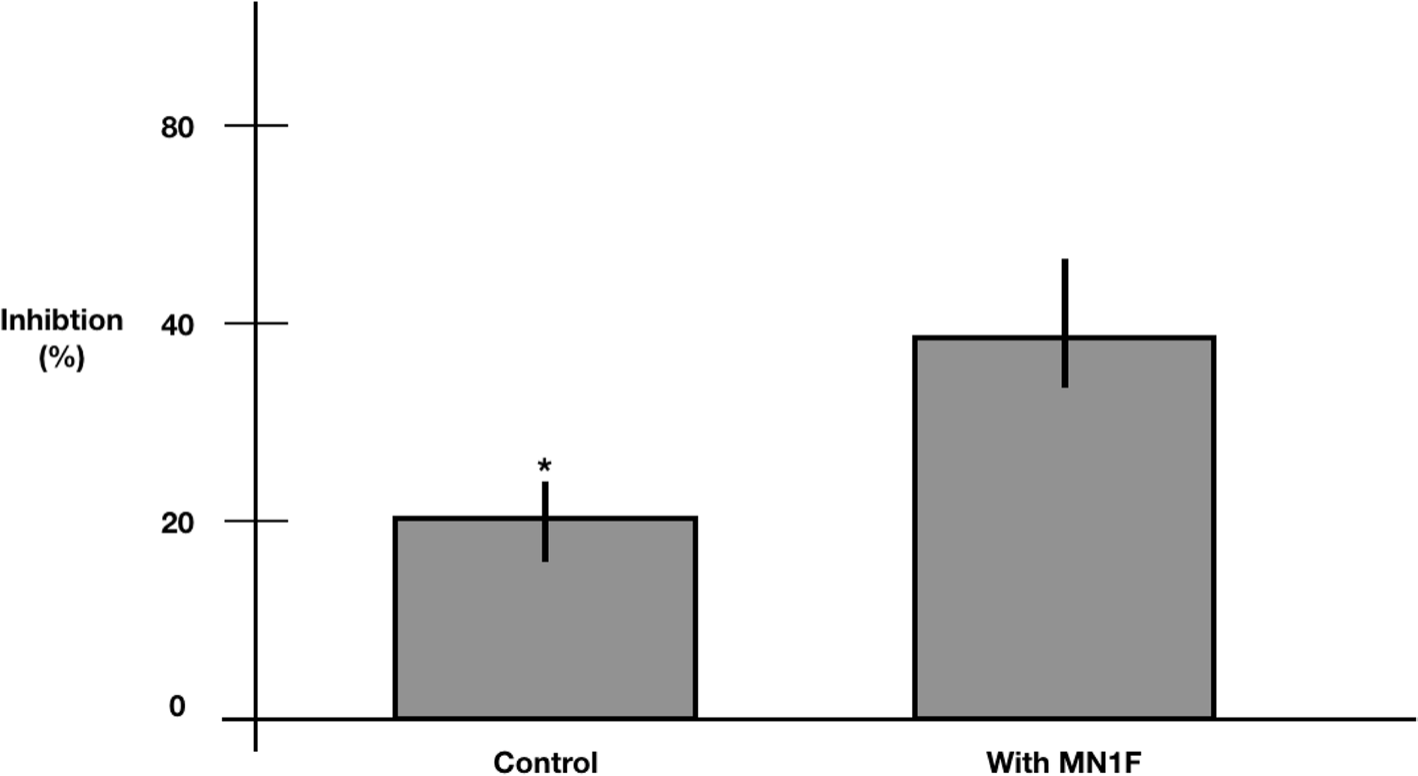
Inhibition in uredospore germination promoted directly by MN1F contact. * The asterisk indicates statistically significant differences (Tukey, α = 0.05)

The MN1F strain showed ability to inhibit 40% of uredospores germination, when applied as a solution in the same concentration of in vitro conditions (Fig. 7). Therefore, this effect over the germination, which is essential for the infection process, may be related with the control of the disease in the coffee seedlings in the treatment ANTP. However, because the coffee seedlings treated with MN1F showed lower incidences of coffee rust until the last evaluation (54 days after the inoculation), probably other mechanisms of control, such as induction of resistance, may also be acting in the biocontrol of the disease.

## Discussion

The phyllosphere is reported to be a harsh environment to colonize because it imposes numerous abiotic stresses on the microbial community: variations in temperature, low availability of nutrients and water, the effect of winds and rain and UV radiation [19, 20]. Taking it into account, we searched for genes in the genome of MN1F that would help to explain its leaf surface colonizer behavior, especially with respect to tolerance to abiotic stresses.

For instance, genome analysis indicated that MN1F has several heat-shock and cold-shock proteins, which are consistent with an environment where the temperature can abruptly change [20]. Genes related to osmotic/drought stress were also found, either by controlling the proline/betaine balance [21] or by controlling the accumulation of glycerol that helps with environmental osmo-adjustments [22]. In addition, the presence of Aquaporin Z, which also acts in freezing tolerance [23], suggests that MN1F had several means of dealing with desiccation. This perception was confirmed in PEG-induced drought stress experiment (Fig. 3), where even at very high concentrations of PEG6000, there was still bacterial presence. EPS synthesis would also assist the biofilm production [24], as well as fimbriae synthesis [25]. The bacterial extracellular matrix built in certain regions of the leaf is crucial to establishment of microbial communities, where the biofilm would serve not only for protection, but also to maximize the search for resources [19, 20, 26].

Another characteristic of MN1F is the degradation network of phenolic compounds that leads to the citrate cycle (Figs. 1 and 2). It is noteworth that some of these compounds have antimicrobial activity, especially salicylate, which mediates the immune response of plants [27]. Therefore, the degradation and incorporation pathways of these phenolics would not only serve as carbon sources in an environment naturally poor in resources [19, 20], but also as protection against the harmful effects of the compounds. In fact, phenolic compounds produced by leaf tissue help to regulate microbial community [27].

Caffeine is a compound abundant in coffee leaves. Although no genes were found for degradation of caffeine, and growth experiments verified no use of caffeine as carbon and nitrogen sources (Figure S2), MN1F has a high tolerance to caffeine (Fig. 4). This data suggests that MN1F has another tolerance mechanism instead of caffeine degradation. It is possible that MN1F has a potential caffeine extrusion mechanism. This high tolerance may have been selected by the leaf environment rich in caffeine, which would give MN1F an advantage to colonize leaves of the coffee tree.

*Pseudomonas* was previously reported as a biocontrol agent for coffee rust [6, 28]. In this work, we showed that MN1F decreased the incidence and severity of coffee rust under laboratory and greenhouse conditions. In fact, in the greenhouse experiments, inoculation with MN1F proved even better than the fungicide in terms of incidence when the bacteria inoculation was done before the inoculation of the pathogen. The loss of effect of the fungicide is a phenomenon observed in coffee crops due to the selection of resistant *H. vastatrix* strains [29]; however, in our experiment we observed that on the 40^th^ and 47^th^ days after the inoculation, the fungicide managed to significantly reduce the damage by the pathogen. From day 47 to day 54 there was a decrease in effectiveness, something that also happened with resistant inductor ASM; although the mechanisms of action of the two products are different. It is suggested that over time, chemical compounds lose their effectiveness, requiring a new application; one of the problems of chemical pest control [30]. On the other hand, in all treatments with MN1F there was a delay in the evolution of the symptoms. The application of the biocontrol agent before the fungus infection (ANTP) was the treatment that gave the most positive results, perhaps because there is an immediate antagonistic effect on the germination of uredospores, hindering the subsequent establishment of the pathogen [6, 28]. This antagonism may be due to the production of some anti-fungal compounds or the rapid occupation of leaf sites for the establishment of bacteria, making it difficult for the fungus to compete for resources [20]. However, the experiment with the application of the filtered supernatant showed that there was no significant effect on *H. vastratrix* infection, which corroborates with the analysis of the genome that predicts that MN1F does not produce anti-fungal compounds as seen in other biocontrol agents. Curiosly, this data is in contrast to Haddad et al., [31], which found that *P. putida* P286 supernatant was equally effective than the bacterial cells at reducing the *H. vastatrix* uredospores germination, most likely through the action of antifungal compounds. Nevertheless, our results suggest that MN1F biocontrol mechanisms are dependent on the direct bacteria/fungus interaction.

We suggest that competition for resources may be one of these mechanisms as well seen for rhizosphere bacteria [32, 33]. Another possibility to be considered concerns the type VI secretion system (T6SS). It is known that this system is linked not only to the virulence of pathogens, but also in the colonization of commensal and beneficial bacteria [34]. It has been discovered that bacteria can use T6SS to deliver effectors with antifungal activity [35]. MN1F has T6SS and also five copies for RHS proteins that can have exotoxin activity in both eukaryotic and prokaryotic targets using T6SS as delivery system [36]. We suggest that, given the need for direct interaction between bacteria and fungi for biocontrol in this model, MN1F could use T6SS as a delivery system for RHS toxins as seen in others *Pseudomonas* with biocontrol activity [37].

Pre-applications of MN1F with two days before *H. vastatrix* infection (INDP) showed good results, but less effective than ANTP. Interestingly when applied before and after infection (ANTP + INDP) the effect is worse when compared to ANTP. We suggest that there may be a competition mechanism between the first population (coming from the first application) and the second population (coming from the second application). In fact, in the rhizosphere the establishment of several different populations of bacteria can spoil the intended biocontrol action [38]. This behavior, added to the fact that the time of application influences the use of MN1F, must be taken into account when planning the application of the bacteria to improve its biocontrol ability.

## Conclusions

Genome analysis and physiological tests showed that *Pseudomonas* sp. MN1F has characteristics consistent with a phyllosphere bacteria, being probably a constant and adapted resident on the coffee leaf. MN1F also showed antagonism against *H. vastatrix*, what indicates this bacterial isolate as a potential biocontrol tool for coffee rust disease.

## Material and methods

### Isolation of *Pseudomonas* spp. MN1F

Leaves of a six-month-old *C. arabica* (cv. Catuaí Amarelo IAC 62) were collected at Fazenda Santa Elisa of the Agronomic Institute of Campinas (IAC, Campinas, Brazil, 22^o^53 'S / 47^o^5' W, 664 m, typical dystrophic Red Latosol soil). No permission was necessary to collect samples at Fazenda Santa Elisa since it contains a coffee germplasm that is part of IAC. The leaves were soaked in 10 mM MgSO_4_.7H_2_O buffer and stirred (3000 rpm) for 15 min. The resulting wash and macerate were used for the dilution series in Petri dishes containing King’s B Medium [39]. The plates were then incubated at 28ºC and the growth of individual fluorescent colonies characteristic of *Pseudomonas* was monitored within 48 h after plating. A colony was isolated and named as MN1F. Bacterial identification was performed using TYGS program (https://tygs.dsmz.de/), which digitally performs a DNA-DNA hybridization and calculates/compares the G + C contents between the studied genome and those deposited in the NCBI [40].

### Genome sequencing, annotation and functional analysis

DNA from MN1F was extracted with Trizol® reagent (Life Technologies Corporation, Carlsbad, CA, USA) according to the manufacturer’s specifications. DNA was sequenced using the Illumina MiSeq platform at the Carolina Center for Genomic Sciences (CCGS) at the University of North Carolina (UNC), Chapel Hill. Following quality check, 300-bp PE libraries were produced to determine the orientation and relative position of the contigs produced by sequencing. The libraries were constructed using the TruSeq sample preparation kit (Illumina). The assembly of the reads was carried out with Velvet assembler [41]. Phylogenomics was performed using the Type Strain Genome Server—TYGS program (https://tygs.dsmz.de/) [39]. Gene prediction was completed using GeneMark and Glimmer [42, 43]. The functional and comparative annotation of bacterial genomes was performed using the RAST program according to the developers’ specifications [44]. Metabolic maps were drawn based on the KEGG tool [45]. Mobile elements were identified using ISFinder (https://www-is.biotoul.fr/) according to Siguier et al. (2006) [46]. The potential production of secondary metabolites was examined using antiSMASH 2.0 software according to Blin et al. (2013) [14].

### EPS production and caffeine tolerance

To examine EPS (exopolysaccharide) production in response to water stress, bacteria (MN1F and *E. coli* as a control) were grown in liquid King’s B medium supplemented with 35 mM, 45 mM, and 55 mM polyethylene glycol (PEG 6000). EPS determination was performed according to the protocol described by Papinutti [47]. For caffeine tolerance test, MN1F were grown in basal liquid medium (2.3 g KH_2_PO_4_, 2.9 g Na_2_HPO_4_.2H_2_O, 1.0 g NH_4_Cl, 0.5 g MgSO_4_.7H_2_O, 0.5 g NaHCO_3_, 0.01 g CaCl_2_.2H_2_O) supplemented with different caffeine concentrations (0.8, 2, 3, 6, 10, 15 g/mL). After 8 h of growth, a 10 µl aliquot was taken from the medium and plated in basal medium supplemented with 1,5% agar. Bacterial viability was determined as the number of colony-forming units (CFU/mL).

### Use of caffeine as sources of carbon and nitrogen

In order to verify the ability of MN1F to incorporate caffeine as a source of carbon and nitrogen, bacteria were grown in basal liquid medium (2.3 g KH_2_PO_4_, 2.9 g Na_2_HPO_4_.2H_2_O, 1.0 g NH_4_Cl, 0.5 g MgSO_4_.7H_2_O, 0.5 g NaHCO_3_, 0.01 g CaCl_2_.2H_2_O) with glucose and NH_4_Cl as primary sources of carbon and nitrogen, with concentrations ranging from 0 to 2 g/L for NH_4_Cl and from 0 to 8 g/L for glucose, fixing the caffeine concentration (1 g/L). OD_600_ was measured at each stipulated time point (0, 10, 20, 30, 40, 50, 60 and 70 h post bacterial inoculation).

### Degradation and incorporation of phenolics

Tests for degradation of aromatic phytochemicals, which are commonly found in coffee such as benzoate, quinate, cinnamate and salicylate were carried out. The first round of growth was carried out in solid medium with the following composition: 0.2 g/L K_2_HPO_4_; 1 g/L NaCl; 0.15 g/L CaCl_2_ 2H_2_O,; 0.4 g/L MgCl_2_ 6H_2_O,; 0.5 g/L NH_4_Cl,**;** 0.5 g/L KCl,; 0.5 g/L cysteine-HCl,; 0.5 g/L yeast extract,; 5 mM cinnamate or benzoate or salicylate or quinate as the primary source of carbon and agar. A second round of cultivation, now in a liquid medium, was carried out under the same conditions described previously with 5 mM cinnamate or benzoate or salicylate or quinate, which would be degraded as the acetate concentration increased. Phytocompounds were measured with the HPLC analytical column (Agillent) Waters µBondapak C18 (3.9 × 300 mm, 10 µm) and detected in an API-5000 Triple Quadruple mass analyzer. The ESI (-)—MS was performed at adjusted voltages of -4000 V and 70 V.

### Preparation of the microorganisms for biocontrol essays

MN1F was grown in 250 mL of LB medium at 28 °C during 12 h. The inoculum was slightly centrifuged to allow the pellet to be collected without destroying the cells and resuspended in saline (0.9% NaCl) until it reached a concentration of 10^8^ cells per ml. This suspension was used for spray on the leaves. The *H. vastarix* uredospores were collected by scraping the abaxial region of the leaves one day before the experiment. The uredospores were packed in closed eppendorfs and placed in the refrigerator until the experiment. The final concentration of the inoculant was 0.02 g of uredospores per liter of saline (0.02 g/L).

### In laboratory verification of antagonistic potential and effect of extracelular metabolites on infection progress

For checking the direct antagonism and secreted metabolites effect on *H. vastarix* infection, we used Shiomi et al.protocol (2006) [28] with modifications. MN1F was grown under the same conditions above mentioned. The medium was passed through a polycarbonate membrane filter with a pore size of 0.2 µm (Sterlitech®). The filtrate was used immediately for the antagonism experiment. Young leaves of one-year old plants (*C. arabica* IAC 62) with no signs of disease or injury were collected. Discs of 2 cm in diameter were cut. Nine discs were placed in petri dish (the leaf with the abaxial part upwards), each one representing one treatment. The plates contained a germination paper pad that remained moist to accommodate the discs. The treatments used were as follows:

- I- saline + uredospores applied simultaneously.
- II- King’s B medium + uredospores applied simultaneously.
- III- supernatant filtered + uredospores applied simultaneously.
- IV- MN1F + uredospores applied simultaneously.
- V- MN1F + uredospores applied 24 h later.
- VI- Uredospores + MN1F applied 24 h later.
- VII- MN1F only.

Twenty-five uL of MN1F, uredospores, saline and supernatant were applied to the abaxial part of the discs that were incubated in the dark for 24 h. After this period, the plates were left incubated in 12 h of photoperiod, 500–1000 lx, 22 ± 2 °C, and approximately 100% relative humidity. Thirty days later, the severity of the lesions (measured by the percentage of injured area) was evaluated using images of leaves digitized by a digital camera and estimated using Téllez et al., approach [48]. The experimental design was randomized blocks (*n* = 3), each replicate consisting of three leaves.

### Greenhouse antagonism evaluation

After checking the antagonism in detached leaves, plants of *C. arabica* IAC 62 of 6 months old, were used for greenhouse experiments. Six treatments were carried out:

- Control- Infected plants without treatment.
- ASM- Acibenzolar-S-methyl (plant defense inducer analogous to salicylic acid) formulated as BION 500 WG® containing 50 g/kg of active ingredient (Syngenta). Plants were sprayed two days before *H. vastatrix* inoculation with a 0.01 g/L solution of ASM..
- Fungi- Fungicide Priori Xtra (containing 200 g/L of azoxystrobin and 80 g/L of cyproconazol). Plants were sprayed two days before *H. vastatrix* inoculation,with a 1.25 ml of commercial product per liter of water.
- INDP- Plants inoculated with MN1F two days before *H. vastatrix* inoculation.
- ANTP- Plants inoculated with MN1F a few minutes before *H. vastatrix* inoculation.
- ANTP/INDP- Plants were inoculated two days before and a few minutes before the inoculation with MN1F.

Uredospores were inoculated by high pressure spray. Plants were placed in a dark humid chamber (near 100% humidity) for 48 h. Each treatment contained 16 plants and the first three pairs of leaves from bottom to top were evaluated at each evaluation. Three evaluations were performed after the appearance of the first symptoms at 40, 47 and 54 days after inoculation. The incidence was estimated using the formula (Number of leaves with lesions / Total number of leaves) × 100. The average number of injuries per plant was also evaluated. The trial was set up in a randomized blocks with 6 treatments, each one consisting of sixteen plants.

### Effect of MN1F on uredospore germination

The suspension of bacteria and fungi used previously was used for the uredospore germination inhibition test. The substrate used was potato-dextrose-agar. The effect of the suspension of bacteria was evaluated after 8 h, in the dark and at a temperature of 24 °C. Saline solution 0.9% NaCl was used as control. Microphotographs were taken from the uredospores with a digital camera (AxioCam ICc1) mounted on a compound microscope (Carl Zeiss, Primo Star). For each treatment, 3000 uredospores were selected, and the number of germinated spores was recorded. The experiment was performed in triplicate and an analysis of variance was performed to verify if there was a difference between treatments. The percentage of inhibition of uredospore germination was calculated with the formula: I = [(C—D)/T] × 100, where I = percentage of non-germinated uredospores; C = number of germinated uredospores in the control treatment; T = number of germinated uredospores in the experimental treatment.

### Statistical analysis

The analysis of variance (ANOVA) of the treatments was carried out using software SPSS 19.0. For physiological experiments, Tukey test (alpha = 0.05) was applied. For biocontrol experiments, Dunnett test (*p* < 0.05) was applied.

## Supplementary Information

**Additional file 1:** **Figure S1.** Phylogenetic tree containing MN1F and other Pseudomonas species. Phylogenetic tree generated in TYGS using the FastME 2.1.6.1 program present on the website. 100 replications were used, with an average branch support of 89.6% according to the developer’s specifications. **Figure S2.** Measurement of caffeine incorporation as carbon and nitrogen source. Experiment to verify if MN1F was able to use caffeine as a source of carbon and nitrogen. Primary sources were removed and caffeine was offered as an alternative source. The intention was to induce cells to “replace” glucose and NH4Cl with caffeine as sources of carbon and nitrogen respectively, which did not occur. **Figure S3.** Sketch summarizing the set-up of leaf disc experiment of infection with *H. vastatrix*. **Table S1.** MN1F insertion sequences detected by the ISFinder web tool. *Pseudomonas syringae* was the main IS donor with 34 copies.

## Data Availability

The generated nucleotide sequence of the Pseudomonas MN1F can be accessed in GenBank under accession ASM939188v1 (https://www.ncbi.nlm.nih.gov/assembly/GCF_009391885.1). The datasets generated and/or analyzed during the current study are available from the corresponding author on reasonable request.
