# Supplementary material for: Functional genomics analysis of a phyllospheric Pseudomonas spp with potential for biological control against coffee rust
Source: BMC Microbiol. 2022 Sep 22;22:222. doi: 10.1186/s12866-022-02637-4 (PMC9494895; doi:10.1186/s12866-022-02637-4)
Supplement: Supplementary file 1 — Additional file 1: Figure S1. Phylogenetic tree containing MN1F and other Pseudomonas species. Phylogenetic tree generated in TYGS using the FastME 2.1.6.1 program present on the website. 100 replications were used, with an average branch support of 89.6% according to the developer’s specifications. Figure S2. Measurement of caffeine incorporation as carbon and nitrogen source. Experiment to verify if MN1F was able to use caffeine as a source of carbon and nitrogen. Primary sources were removed and caffeine was offered as an alternative source. The intention was to induce cells to “replace” glucose and NH4Cl with caffeine as sources of carbon and nitrogen respectively, which did not occur. Figure S3. Sketch summarizing the set-up of leaf disc experiment of infection with H. vastatrix. Table S1. MN1F insertion sequences detected by the ISFinder web tool. Pseudomonas syringae was the main IS donor with 34 copies. [file 12866_2022_2637_MOESM1_ESM.doc]

**Supplementary information**

**Figure S1**

**Phylogenetic tree containing MN1F and other Pseudomonas species.** Phylogenetic tree generated in TYGS using the FastME 2.1.6.1 program present on the website. 100 replications were used, with an average branch support of 89.6% according to the developer's specifications.


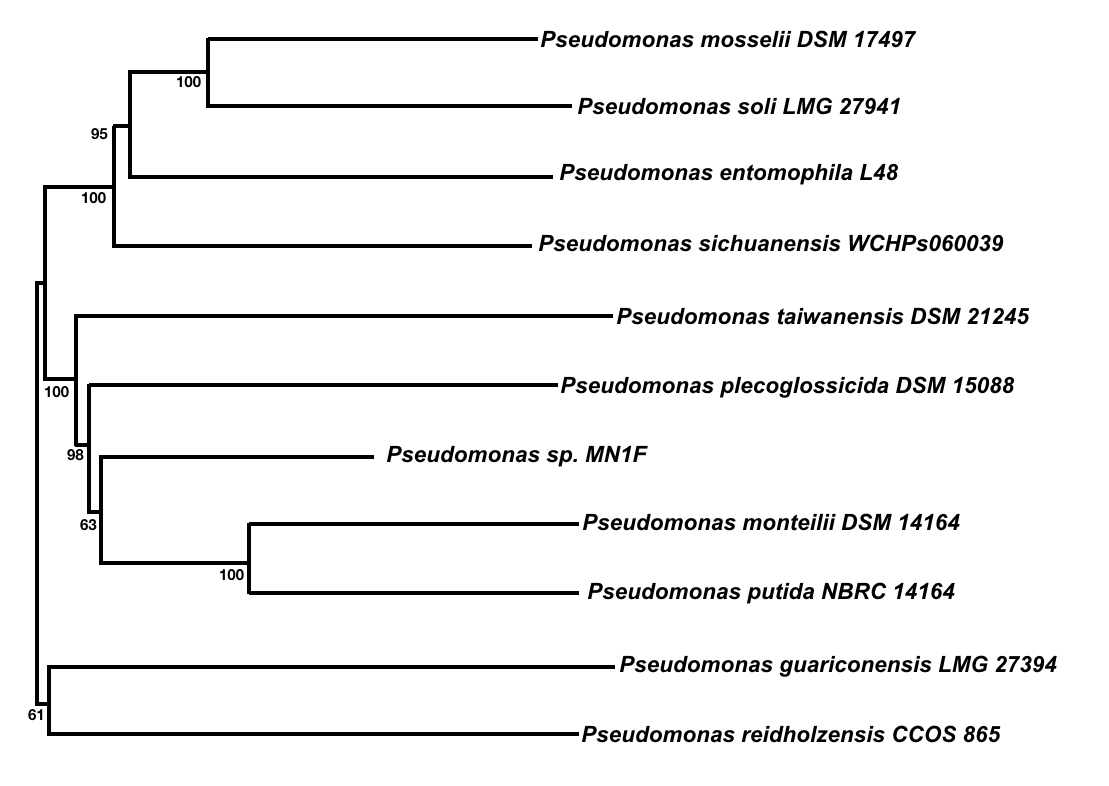


**Figure S2**

**Measurement of caffeine incorporation as carbon and nitrogen source.** Experiment to verify if MN1F was able to use caffeine as a source of carbon and nitrogen. Primary sources were removed and caffeine was offered as an alternative source. The intention was to induce cells to “replace” glucose and NH4Cl with caffeine as sources of
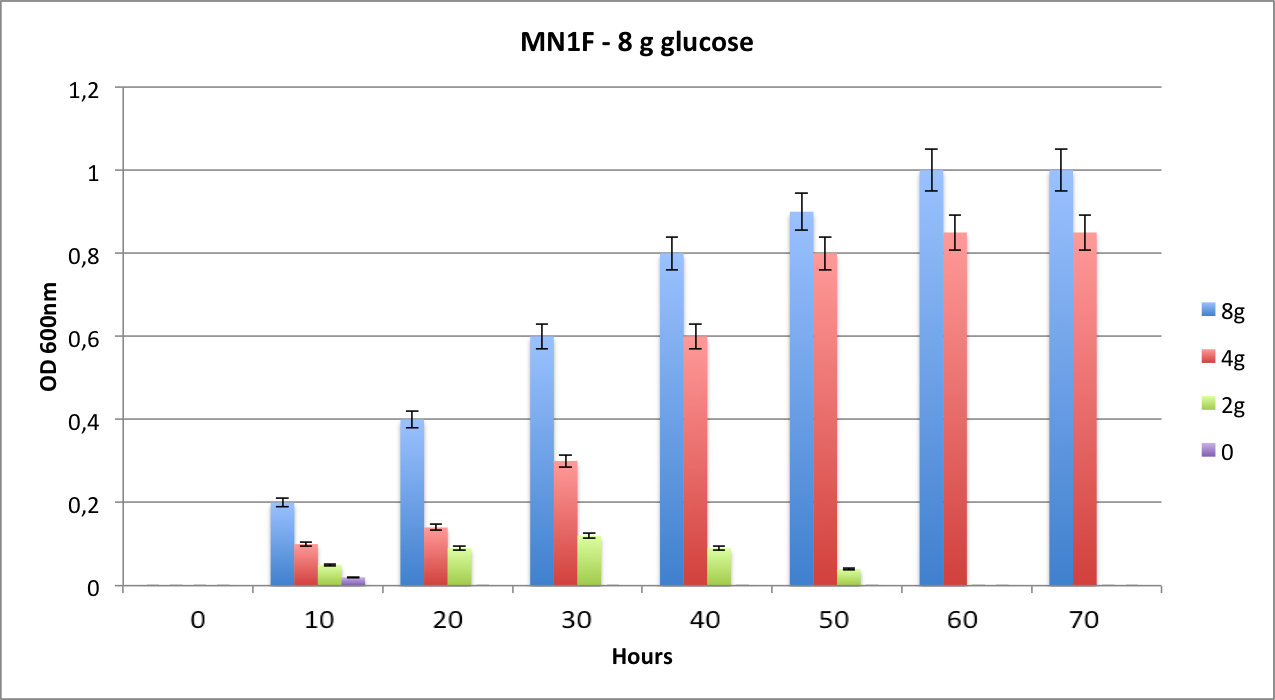
carbon and nitrogen respectively, which did not occur.


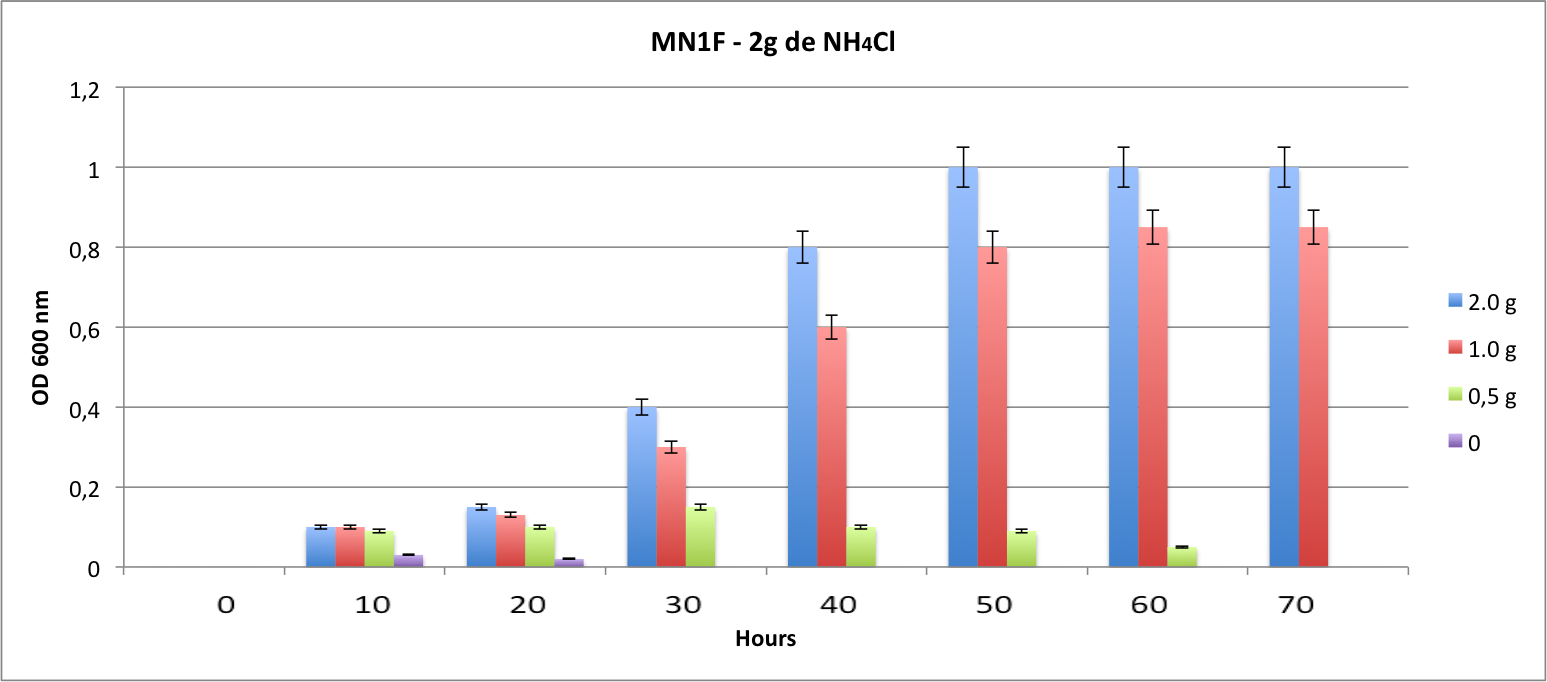


**Figure S3**

**Sketch summarizing the set-up of leaf disc experiment of infection with *H. vastatrix*.**


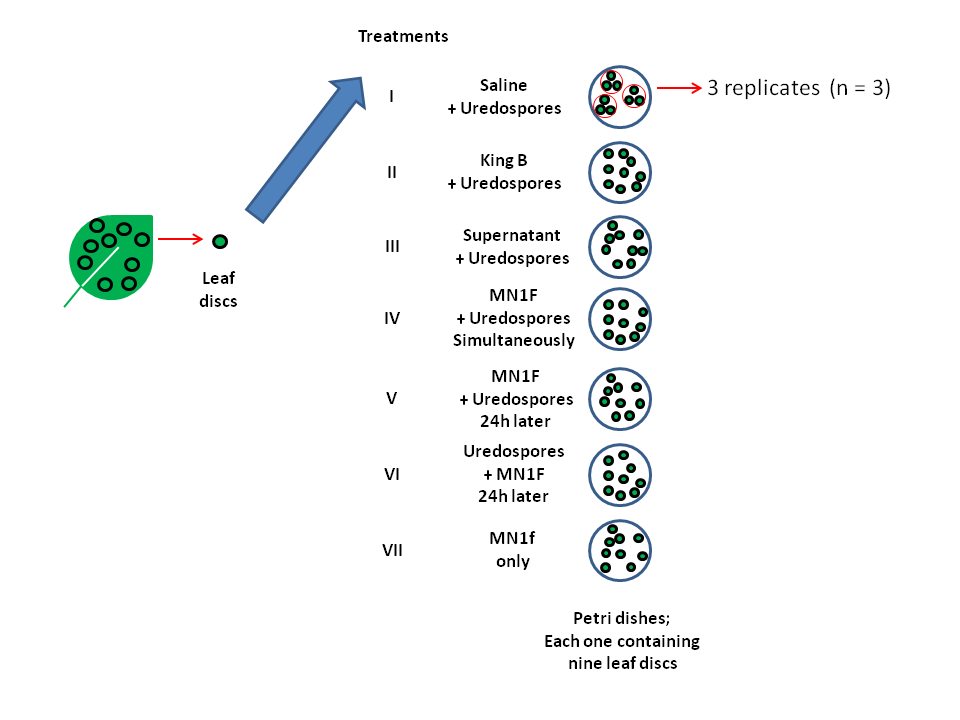


**Table S1**.

MN1F insertion sequences detected by the ISFinder web tool. *Pseudomonas syringae* was
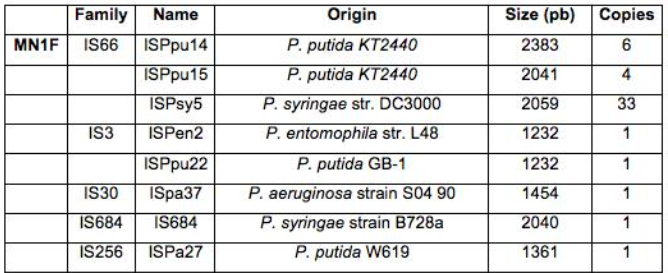
the main IS donor with 34 copies
